# Social predictors of breastfeeding and the impact of interventions on breastfeeding of preterm infants: A longitudinal study

**DOI:** 10.18332/ejm/174125

**Published:** 2023-12-20

**Authors:** Michaela Abrmanová, Iva Brabcová, Valérie Tóthová, Martin Červený

**Affiliations:** 1 Institute of Nursing, Midwifery and Emergency Care, Faculty of Health and Social Sciences, University of South Bohemia in Ceske Budejovice, Česke Budějovice, Czech Republic

**Keywords:** breastfeeding, longitudinal study, infant nutrition, preterm infants, social predictors, interventions

## Abstract

**INTRODUCTION:**

The multifaceted benefits of breastfeeding for mothers and infants include enhanced neurodevelopment and immune function in preterm infants. However, more research is needed to understand the unique factors affecting breastfeeding practices in preterm infants. This study aimed to identify key social predictors of breastfeeding in preterm infants and assess the effectiveness of specific interventions on their feeding practices during the first six months postpartum.

**METHODS:**

A prospective, monocentric, longitudinal study involving a cohort of 201 preterm infants was executed at the Neonatology Department, Ceske Budejovice Hospital, Czech Republic, from January 2020 to January 2023. The STROBE guidelines were used.

**RESULTS:**

The study results elucidated a transition from breastfeeding to bottle feeding and formula within the infants' first six months. Notable social predictors of breastfeeding encompassed factors such as the number of children in the household, the mother's marital status, and the nature of housing. Certain interventions, including immediate skin-to-skin contact between mother and child, and initiation of nutritive feeding within the first half-hour post-birth, significantly influenced the probability of breastfeeding.

**CONCLUSIONS:**

The data underscored that social predictors and nursing interventions substantially shape the breastfeeding practices of preterm infants during the first six months postpartum. Inequities in health outcomes among premature infants can be effectively curbed through comprehensive care models that account for socioeconomic factors influencing breastfeeding.

## INTRODUCTION

It is generally accepted that human breast milk is the optimal source of infant nutrition, especially for premature babies^1^. The World Health Organization^2^ recommends starting breastfeeding within the first hour after birth and maintaining exclusive breastfeeding until the child reaches six months. From the results of a meta-analysis by Victora et al.^3^, it is clear that exclusive breastfeeding is associated with: 1) protection against childhood infections and orthodontic defects, 2) increased intellectual abilities, and 3) reduced risk of being overweight and developing diabetes. According to WHO^4^, one in ten infants globally is born before the 37th week of pregnancy. The same prevalence (about 10%) was reported by Vogel et al.^5^. In the Czech Republic, approximately 7200 babies are born before the 37th week of pregnancy, corresponding to about 6.7% of all births^6^. In 2011, the percentage was 8%, corresponding to the current European average^7^. Healthcare efforts in Europe are concentrated on improving the survival of premature newborns with immature vital functions^4^. However, there is a lack of systematic, comprehensive follow-up care for premature babies and their families. Promoting breastfeeding and providing follow-up care for the child, mother, and the whole family is not about providing purely medical and nursing care but includes comprehensive interdisciplinary care as well. Raising awareness of this issue will have positive societal and economic impacts^8^. Newborns must receive the highest quality nutrition, ideally in the form of breast colostrum^9^. Despite the recognized benefits of breastfeeding, its prevalence declines during the first 12 months after birth. Although the initial breastfeeding rate is promisingly high, there is a significant decrease in the percentage of breastfeeding in the first weeks after the start of breastfeeding, and exclusive breastfeeding becomes increasingly uncommon with time^10^. Breastfeeding in premature babies is often problematic for several reasons. Increasing breastfeeding efficacy can serve as a promising intervention to empower mothers of premature babies and help them overcome barriers linked to breastfeeding prematurity^11^. Factors affecting the breastfeeding of premature babies need to be investigated so that health services can adequately respond to this issue. In the Czech Republic, systematic education about lactation and breastfeeding is crucial for promoting the health of both mother and child. Prospective mothers are provided with comprehensive information in prenatal clinics from gynecologists and midwives, and support through various platforms to ensure successful breastfeeding practices. This education encompasses the benefits of breastfeeding, techniques, common issues, and their solutions. The Czech Republic has a network of prenatal counseling centers where mothers are prepared not only for pregnancy and physiological childbirth but also for breastfeeding the child, underscoring the importance of national awareness and preparation in the areas of lactation and breastfeeding. This article emphasizes the importance of education and consistent support for mothers, which play a key role in informed decision-making and effective breastfeeding practices, focusing on the overall well-being and health of both mother and child. The detailed analysis provides valuable insights contributing to a deeper understanding of the dynamics of maternal and child health in the Czech Republic. The introductory part of the research includes information about education and support in the field of lactation and breastfeeding in the Czech Republic. Education, nationality, and the social background of mothers are key socioeconomic factors influencing breastfeeding practices. The research findings reveal common sociodemographic patterns among mothers, with those who are married, employed, and living in family houses or apartments, having a significantly higher probability of breastfeeding. In recent years in the Czech Republic, approximately 7% of children are born prematurely, meaning before week 37+0 of pregnancy, out of the total number of newborns. Of this number, around 2% are born very prematurely, that is, before the 32nd week of pregnancy^6^. The aim of the study was to identify social predictors of breastfeeding and the influence of interventions on the nutrition of premature babies during the first six months after birth.

## METHODS

### Study design

This was a prospective monocentric longitudinal study. The research was conducted at the Neonatology Department, Ceske Budejovice Hospital, Czech Republic. The neonatology department consists of a unit for the care of physiological newborns, an intensive care and resuscitation unit, two intermediate care units, a developmental outpatient clinic unit, and a breast milk bank. The research was conducted between January 2020 and January 2023.

In our study, we categorized preterm birth based on the duration of the pregnancy into four distinct subgroups as delineated by the reference^12^. Infants born before the completion of week 28 (meaning less than 28+0) were classified as ‘extremely preterm’. Those born between weeks 28+0 and 31+6 were categorized as ‘very preterm’. Births that occurred between weeks 32+0 and 33+6 were considered ‘moderately preterm’. Finally, infants born from week 34 up to 36+6 were designated as ‘late preterm’.

### Inclusion criteria

The research included 201 premature babies born after the end of the 32nd week and before the end of the 37th week of gestation. Only premature babies with issues more or less related to their immaturity were included in the study. The frequency of these births in the selected hospital was approximately 40–50 per year. In Ceske Budejovice Hospital, a unique approach to caring for mothers with premature babies is offered, emphasizing the importance of continuous contact between the mother and the child. This approach, known as ‘rooming in’, allows mothers to stay with their children in the mother and child unit, which is highly encouraged to promote regular ‘skin-to-skin’ contact. This close contact is key to supporting the bond between mother and child, and has many proven benefits including supporting breastfeeding, stabilizing the heart rate and breathing of the child, reducing stress and pain, and aiding the thermoregulation of premature babies.

### Exclusion criteria

Children were excluded if they had: severe congenital malformations, gastrointestinal surgery, severe cranial morbidity (e.g. major central nervous system hemorrhage), severe pulmonary morbidity (i.e. severe bronchopulmonary dysplasia), and life-threatening conditions.

### Measures

*Independent sociocultural variables*

The sociocultural demographic data of mothers were documented such as age, nationality, marital status, education level, employment category and type of work before current maternity leave, number of family members, and place of residence. We also documented the mother’s medical history, the course of the last pregnancy, and childbirth. We noted whether the birth was spontaneous or cesarean, and the number of: pregnancies, births, children, and abortions.

*Independent intervention variables*

Interventions included lactation support and breastfeeding, in which we monitored whether the first attachment of the newborn to the breast took place within the first half hour after birth, skin-to-skin contact between mother and child in the delivery room, non-nutritive attachment to the breast in the delivery room, and whether there was early transfer of colostrum from mother to child, no later than two hours after birth. We also noted whether the mother received information from a lactation consultant, whether orofacial stimulation took place during hospitalization, and if there was regular mother–child skin-to-skin contact during hospitalization. We further noted whether babies were breastfed with only breast milk, or received breast milk or infant formula through a tube. Methods such as gravity feeding or administering nutrition from a beaker were also documented. Additionally, we noted whether newborns were fed breast milk only, a combination of artificial formula and breast milk, or artificial formula only.

### Dependent variables

The method of feeding premature babies on days 7, 14, and 21, and in the 3rd and 6th months of life was monitored.

### Data collection

Data regarding premature babies were collected from medical records during their hospitalization. After being released into home care, premature babies were monitored within the neonatal developmental outpatient clinic – Development Care Center of the Ceske Budejovice Hospital. Personal contact was established with most mothers, and the mothers then communicated information voluntarily by phone, at-home meetings, or online. The study used repeated follow-up of children from birth until they were six months of age. Bias errors were minimized by checking data correctness in 10% of records. In this context, 250 mothers were approached with the opportunity to participate in the study. They were informed about the research protocol, data collection methods, and potential benefits and risks of participation. After providing all relevant information, 201 respondents agreed to participate in the study, and their children were subsequently included in the research. Obtaining informed consent was crucial for the ethical integrity of the entire research process.

### Ethical approval

Only children whose mothers agreed to have their medical records viewed and agreed with the research were included in the research. The study was approved by the Ethics Committee of the South Bohemian University Faculty of Health and Social Sciences, on 11 October 2020 in accordance with the Declaration of Helsinki^13^. Consent for research at the Ceske Budejovice Hospital was approved on 25 October 2020.

### Data analysis

We used the IBM SPSS version 28.0 to process the data. The descriptive statistics included frequencies (absolute and relative), mean with standard deviation, and range (minimum, maximum). We present median and mode where appropriate (in the case of skewed data). To test the relationship between dependent and independent variables, a logistic regression model with the function of transforming logit for expressing chances (odds ratio, OR) was chosen. When using categorical variables in regression, differences in probabilities were compared to the reference category. Two regression models aiming at the explanation of the occurrence/frequency of breastfeeding were studied: one involved four demographical descriptors (number of children, sex of the child, marital status, place of residence), and the other included five nursing interventions. No other adjusting variables were used in either of the models due to the limited sample size. The significance level was set at p<0.05.

The reliability of breastfeeding interventions was tested by calculating Cronbach’s alpha coefficient. The lower value for the acceptable confidence level was 0.6, and the upper value was 0.9. The interventions supporting breastfeeding had a Cronbach alpha value of 0.702. The reliability of the monitored variables was sufficient.

## RESULTS

Approximately three-quarters of the monitored mothers had Czech nationality (74.6%), one-sixth were of Roma nationality (16.9%), and the rest were of other nationalities. In terms of education, a quarter of the mothers had secondary education without an A-level examination (25.4%), an eighth of the mothers had only primary education (12.4%), and the remaining were divided equally between secondary (with A-level) and university education. In all, 60.1% of the mothers surveyed were employed before giving birth; the second most common category was unemployed (17.9%), followed by housewives (8.0%), part-time employed (6.0%), self-employed (5.5%), student/apprentice (1.5%), and occasional work (1%). Neither manual nor mental work predominated in the research group.

The most common relationship status was married (65.7%), followed by unmarried cohabitation (19.4%), single (11.9%), and divorced (3.0%). The percentage of natural births to cesarean section was 44.8% versus 55.2%, which was skewed compared to the population of full-term newborns. This is consistent with the perinatal development of premature babies, where complications lead not only to a premature birth but often also to acute cesarean section (Supplementary file Table 1).

The mean age of mothers was 31.6 ± 4.9 years. The youngest mother in the group was aged 16 years, and the oldest was aged 45 years. Most were first-time mothers; however, it ranged all the way to nine prior pregnancies (including three miscarriages). The average number of children per mother was 2.1 ± 1.2. The highest number was seven children. The smallest newborn weighed 1410 g, and the heaviest 3320 grams; the average birth weight was 2215.4 ± 345.9 g. The mean gestational age of the newborns was 245.9 ± 9.7 days. The average weight of the children at six months was 6720 g, and the average duration of hospitalization was 13 days (Table 1).

**Table 1:** Personal history of mothers and their children, 2020–2023 (N=201)

	*Mode*	*Median*	*Mean ± SD*	*Min*	*Max*
Maternal age (years)	30	28	31.6 ± 4.9	16	45
Number of pregnancies	1	2	2.1 ± 1.2	1	9
Number of children	1	1	2.1 ± 1.2	1	7
Number of miscarriages	0	1	0.4 ± 0.6	0	3
Number of family members	3	4	3.9± 1.2	1	9
Length of pregnancy (days)	252	246.0	245.9 ± 9.7	224	260
Birth weight of children (g)	2420	2250	2215.4 ± 345.9	1410	3320
Weight of children at 6 months (g)	7000	6720	6797.8 ± 952.9	5000	9600
Duration of hospitalization (days)	14	13	14.7 ± 6.7	6	39

Table 2 provides an overview of interventions that support breastfeeding. Almost two-thirds of the children (64.7%) were not attached to the mother’s breast either immediately or shortly after birth. Non-nutritive breast attachment (skin-to-skin contact) occurred in 54.1% of the children. Colostrum was consumed within two hours of birth in 69.7% of the children. More than three-quarters (77.1%) of the children received orofacial stimulation once a day. Only 3.5% of the mothers did not have skin-to-skin contact with their babies at least three times a day; the vast majority (96.5%) had such contact. Education of mothers by a lactation consultant or a specialized pediatric nurse during the hospitalization of the child took place in almost all mothers (98.5%).

**Table 2:** Interventions supporting breastfeeding, 2020–2023 (N=201)

*Placing the baby on the mother’s breast in the delivery room (nutritive attachment)*	*n*	*%*	*Orofacial stimulation of the child during hospitalization*	*n*	*%*
Yes	71	35.3	Once a day	155	77.1
No	130	64.7	Twice a day	27	13.4
			Three times a day and more	12	6.0
			None	7	3.5
**Non-nutritive breast attachment in the delivery room**			**Skin-to-skin contact at least three times a day**		
Yes	109	54.2	Yes	194	96.5
No	92	45.8	No	7	3.5
**Early application of colostrum within 2 hours after birth**			**Education of mothers by a lactation consultant**		
Yes	140	69.7	Yes	198	98.5
No	61	30.3	No	3	1.5

**Figure 1:**
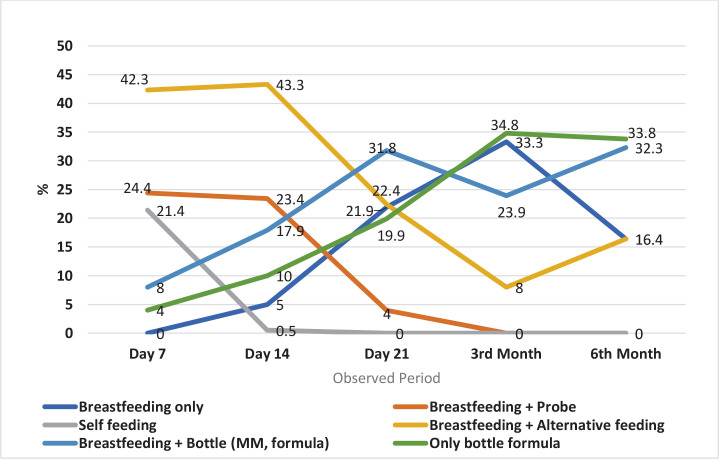
Children's nutrition – development over time (%) – longitudinal study (n 201, 2020-2023)

Figure 1 shows the development of nutrition methods in children from day 7 to six months after birth. At the beginning of the monitored period, the most prominent variant was breastfeeding and alternative milk/formula administration methods. This method was practiced by 42.3% of mothers. However, after a slight increase on day 14 (to 43.3%), there was a decrease in the use of this method. The lowest value was observed at month 3 (8.0%) but rose to 16.4% at the end of the study period. The next two most common feeding methods, breastfeeding combined with tube feeding and gravity feeding showed a continuous decrease during the monitored period; baseline values of 24.4% and 21.4%, respectively, quickly decreased to 0% at three months and day 21, respectively. None of the babies was fully breastfed on day seven. There was a steady increase by the third month to 33.3%, followed by a fall to only 16.4% by the sixth month. The last two variants (which use bottles) were relatively under-represented on day 7, i.e. breastfeeding plus artificial formula administered by the bottle (8.0%) and bottle-only formula (4.0%). Both categories increased in the monitored period, and despite fluctuations in months 3 and 6, they were the most common at the end of the study period, with 32.3% and 33.8%, respectively. Thus, at the end of the period, in month 6, two-thirds of babies are bottle-fed with formula. The remaining third of the babies were at least partially breastfed at 6 months, i.e. half were fully breastfed, and the other half were breastfed plus alternative supplementation.

Figure 1 shows the evolution of the pattern of feeding of the newborns and infants studied from day 7 to month 6. At the beginning of the follow-up period, most infants (42.3%) were breastfed with alternative complementary feeding, while another large group of infants (24.4%) was breastfed with complementary feeding with a tube. Only slightly fewer children (21.4%) were self-fed. The smallest proportion of infants fall into the group breastfed with bottle or exclusively bottle fed (12.0%), and none of the infants was fully fed at day 7. The trend over time shows a change in the representation of each category. The most represented categories, which together account for almost 90% of the observed children, account for only 16.4% of the cases at the end of the follow-up period; these are children breastfed with complementary alternative feeding. Conversely, the least represented categories increase in importance at the beginning of the follow-up period and account for two-thirds of all cases at 6 months of age; these are bottle-fed children either fully or in combination with breastfeeding. It is interesting to look at the proportion of children who were fully breastfed. No child was fully breastfed at day 7, but the proportion rises until month 3, when it is the second most represented category (33.3%), but by month 6 the proportion falls to 16.4%. Although most children were bottle-fed with formula at the end of the period, the picture of the proportion of children who were at least partially breastfed is promising. Almost two-thirds of children (65.1%) were either fully or at least partially breastfed (with bottle supplementation or alternatives).

### Social predictors of breastfeeding in a sample of premature babies

With more children in the family, the chances of breastfeeding a premature baby decreased throughout the monitored period. On the seventh day, the chance of single mothers and mothers in cohabitation relationships breastfeeding was statistically less than that of married mothers. Marital status was not a factor in the rest of the monitored period. The sex of the baby also affects breastfeeding. Girls were almost twice as likely as boys to be breastfed. Women who lived in a family house or apartment were two to three times more likely to be breastfeeding on day 21 as well as at six months (Table 3).

**Table 3:** Influence of social characteristics on breastfeeding (binary logistic regression - Model 1), 2020–2023 (N=201)

*Variables*	*Breastfeeding day 7*			*Breastfeeding day 14*			*Breastfeeding day 21*			*Breastfeeding 3rd month*			*Breastfeeding 6th month*		
	*AOR*	*95% CI*	*p*	*AOR*	*95% CI*	*p*	*AOR*	*95% CI*	*p*	*AOR*	*95% CI*	*p*	*AOR*	*95% CI*	*p*
**Number of children**	0.6	0.4–0.9	0.010*	0.6	0.4–0.8	0.002**	0.7	0.5–0.9	0.042*	0.6	0.4–0.8	0.002**	0.6	0.4–0.9	0.012*
**Sex of the child**															
Boy (Ref.)	1			1			1			1			1		
Girl	1.2	0.5–3.2	0.730	1.6	0.8–3.2	0.161	1.9	1.0–3.7	0.029*	1.3	0.7–2.5	0.368	1.3	0.7–2.5	0.408
**Marital status**															
Married (Ref.)	1			1			1			1			1		
Divorced	-	-	0.999	2.4	0.2–24.7	0.466	0.9	0.2–5.5	0.997	1.8	0.3–10.2	0.506	1.6	0.3–9.2	0.574
Unwed	**0.2**	0.1–0.7	0.015*	0.6	0.2–1.7	0.015*	0.8	0.3–2.2	0.646	1.3	0.5–3.6	0.639	0.8	0.3–2.4	0.686
Living with a partner	**0.2**	0.1–0.7	0.010*	0.8	0.3–1.8	0.010*	0.8	0.4–1.8	0.610	0.8	0.4–1.8	0.598	0.6	0.3–1.5	0.273
**Place of residence**															
Block of flats (Ref.)	1			1			1			1			1		
Family or apartment building	0.5	0.1–1.7	0.240	0.9	0.4–2.2	0.240	2.3	0.9–5.5	0.050*	1.7	0.7–3.9	0.228	3.2	1.3–7.7	0.228
Hostel	1.6	0.4–6.1	0.457	1.7	0.8–3.9	0.457	1.6	0.8–3.4	0.189	1.3	0.6–2.7	0.523	1.9	0.9–4.4	0.523

* p<0.05,

** p<0.01,

*** p<0.001.

The chance of breastfeeding of the baby within half an hour after birth increased 4.3 times, 2.5 times on day 21, and 2.3 times at month 3. Conversely, non-nutritive attachment of the baby to the mother’s breast within half an hour after birth statistically significantly reduced the chance of breastfeeding. Regular contact between mother and child increased the chance of breastfeeding ten-fold, namely on day 7 and day 14. Early consumption of colostrum after birth and orofacial stimulation of the child during hospitalization did not increase the likelihood of full breastfeeding (Table 4).

**Table 4:** Effect of interventions on breastfeeding (binary logistic regression - Model 2), 2020–2023 (N=201)

*Variables*	*Breastfeeding day 7*			*Breastfeeding day 14*			*Breastfeeding day 21*			*Breastfeeding 3rd month*			*Breastfeeding 6th month*		
	*AOR*	*95% CI*	*p*	*AOR*	*95% CI*	*p*	*AOR*	*95% CI*	*p*	*AOR*	*95% CI*	*p*	*AOR*	*95% CI*	*p*
**Nutritive attachment of the baby after birth**	4.3	1.3–13.8	0.015*	1.6	0.8–4.2	0.145	2.5	1.1–5.7	0.027*	2.3	1.0–5.3	0.050*	1.5	0.6–3.8	0.334
**Non-nutritive attachment of the baby after birth**	0.1	0.1–0.5	0.003*	0.2	0.1–0.5	0.001**	0.5	0.2–1.1	0.104	0.6	0.2–1.2	0.169	0.4	0.2–1.1	0.090
**Early application of colostrum after childbirth**	1.3	0.4–4.7	0.617	1.6	0.4–2.6	0.916	0.7	0.2–1.9	0.474	1.2	0.6–2.7	0.608	1.3	0.6–3.0	0.524
**Orofacial stimulation**	5.6	0.8–40.3	0.090	1.8	0.3–12.6	0.518	3.9	0.4–36.4	0.236	3.1	0.5–20.2	0.222	2.2	0.4–12.5	0.388
**Contact skin to skin ≥3 times a day**	11.4	1.7–76.6	0.013*	10.2	1.6–64.2	0.014*	4.1	0.4–38.8	0.216	4.0	0.7–24.8	0.131	3.1	0.6–16.9	0.199

* p<0.05,

** p<0.01,

*** p<0.001.

## DISCUSSION

Proper feeding of premature babies is an important component of healthy development. Breast milk contains all the necessary nutrients, vitamins, and other trace elements that support this process. The main objective of this study was to identify social predictors of breastfeeding and to analyze the effect of interventions on the nutrition of premature babies in the first six months of life.

### Choice of feeding methods for premature infants

The study’s results suggest a significant transition from breastfeeding premature infants to bottle feeding and formula during the study period. Initially, breastfeeding combined with alternative feeding methods was the most common, but by the end of the study, two-thirds of the babies were bottle-fed. These findings suggest that breastfeeding was not the preferred method in this cohort. The results of the Zhang et al.^14^ study suggest that mothers, within the analyzed set, recognized breastfeeding as the optimal method of feeding babies. Still, many women reported that breastfeeding was uncomfortable and tiring, and viewed bottle feeding as a better alternative. These results show that many mothers find exclusive breastfeeding during the first six months of life problematic. Wirihana and Barnard^15^ found similar results. Mothers who used bottles and breastfed together expressed a desire for education and access to relevant information but reported a lack of these resources. In this study, women who opted for bottle feeding felt uncomfortable seeking help and felt that bottle feeding was easy and that they already had the necessary knowledge and skills. Mothers who preferred formula strongly believed formula-fed babies are less demanding and calmer, and do not require the mother’s constant presence^16^.

### Social factors determining breastfeeding in a sample of premature babies

The results of this study show that, during the study period, more children in a household reduced the likelihood of breastfeeding. Furthermore, on day 7, single mothers and mothers in cohabitation relationships breastfed statistically significantly less than married mothers. The study by Zachariassen et al.^17^ looked at the factors associated with successful breastfeeding of premature babies. The results showed that mothers with high social status and non-smokers were positively associated with breastfeeding. Conversely, multiple pregnancies were negatively associated with the duration of breastfeeding. In our sample of premature newborns, the sex of the baby impacted breastfeeding, with girls being almost twice as likely to be breastfed than boys. Our findings are in agreement with the research of Shafer and Hawkins^18^. Other research, however, suggests a gender bias in the duration of breastfeeding in favor of boys^19-21^.

### Effect of early mother-child contact on breastfeeding in a sample of premature babies

The results of this study suggest that early nutritional attachment of the baby to the breast (i.e. after attachment, the baby sucks from the breast) significantly increases the chance that mothers will still be breastfeeding at different times. Conversely, non-nutritive attachment of the infant to the mother’s breast (i.e. after attachment to the mother’s breast, the baby does not suck) significantly reduced the chance of breastfeeding. In this study, regular mother–baby skin-to-skin contact also significantly affected breastfeeding. Regular skin-to-skin contact increased the chance of breastfeeding on day seven 11.4 times, and 10.2 times on day 14. Evidence suggests that skin-to-skin contact between mother and baby increases changes in weight, length, and head circumference. Skin-to-skin contact also has a potentially beneficial effect in reducing neonatal morbidity and mortality^22^. During their short stay in the delivery room, Nilsson et al.^23^ researched ways to increase maternal confidence in breastfeeding and to promote effective breastfeeding that meets the needs of both mother and baby, while helping the baby to adapt metabolically and the mother to increase breast milk supply. Their study showed a higher proportion of breastfeeding in the intervention group at six months than the control group. Other evidence shows that skin-to-skin contact, which should ideally begin at birth and continue until the end of the first breastfeeding, is also effective. Women who had skin-to-skin contact were more likely to be still breastfeeding one to four months after birth. Women who practiced skin-to-skin contact were more likely to breastfeed exclusively after discharge from the hospital for: 1) one month after delivery, and 2) from six weeks to six months after delivery. In addition, these women had higher mean breastfeeding efficacy scores. Neonates exposed to skin-to-skin contact were more likely to breastfeed successfully during the first feeding. Women who had skin-to-skin contact after a cesarean section were more likely to breastfeed one to four months after giving birth and to breastfeed successfully. However, lack of evidence prevents assessing whether skin-to-skin contact can improve breastfeeding in subsequent periods after a cesarean section^24^.

### Strengths and limitations

Our study has several limitations that need to be noted. The first limitation is the small sample size, which reduces the possibility of generalization to the entire population. For more accurate results, obtaining a larger and more representative sample is necessary. Another limitation was the limited data collection, i.e. from only one hospital in the Czech Republic. Research should be expanded to include more hospitals within the Czech Republic to obtain more relevant results. This would allow us to better understand the wider context and variability of breastfeeding practices. In addition, a deeper and more extensive analysis of the predictors that affect breastfeeding of premature babies needs to be conducted. The strength of longitudinal research is the ability to track changes over time and identify important factors, such as maternal health, living conditions, or socioeconomic status, that can significantly impact breastfeeding.

## CONCLUSIONS

This study found that breastfeeding, combined with an alternative method of administering breast milk, was the most commonly used method of feeding premature newborns. This practice gradually declined and was the least used by the end of the follow-up. Social predictors that increased the chance of breastfeeding included the number of children in the family, the marital status of mothers, and the type of housing. The results indicate that with a higher number of children, the chance of breastfeeding decreased, single women or women living in cohabitation relationships were less likely to breastfeed than married women, and women living in a family house were more likely to breastfeed than women living in a housing estate.

A statistically important factor for successful breastfeeding was the nutritional attachment of the baby within half an hour after birth. Regular skin-to-skin contact between the infant and the mother was also associated with a higher chance of breastfeeding. We found that nursing interventions and several socioeconomic and demographic factors significantly predicted the likelihood of breastfeeding six months after birth.

## Data Availability

The data supporting this research are available from the authors on reasonable request.
