# Supplementary material for: Social predictors of breastfeeding and the impact of interventions on breastfeeding of preterm infants: A longitudinal study
Source: Eur J Midwifery. 2023 Dec 20;7:44. doi: 10.18332/ejm/174125 (PMC10731748; doi:10.18332/ejm/174125)
Supplement: Supplementary file 1 [file EJM-7-44-s1.pdf]

**Table 1** *Basic socio-demographic information of the mothers in our data set, longitudinal study (n 201, 2020-2023)*					
**Nationality of mothers**	**n**	**%**	**Maternal education**	**n**	**%**
Czech	150	74.6	Basic education	25	12.4
Slovak	7	3.5	High school without graduation	51	25.4
Ukrainian	2	1.0	Secondary school with graduation	59	29.4
Roma	34	16.9	Tertiary professional education	6	3.0
Vietnamese	3	1.5	Higher education	59	29.4
Other	5	2.5	Other	1	0.4
**Total**	**201**	**100.0**	**Total**	**201**	**100.0**
**Economic status before childbirth**	**n**	**%**	**Marital status**	**n**	**%**
Unemployed	36	17.9	Married	132	65.7
Housewife	16	8.0	Divorced	6	3.0
Student, apprentice	3	1.5	Unwed	24	11.9
Full-time employed	121	60.1	In unmarried cohabitation	39	19.4
Part-time employment	12	6.0	**Total**	**201**	**100.0**
Self-employed entrepreneur	11	5.5			
Irregular employment	2	1.0	**Type of work**	**n**	**%**
**Total**	**201**	**100.0**	Intellectual	68	33.8
			Manual	66	32.8
			Combined	40	19.9
**Place of residence**	**n**	**%**	no response	27	13.5
Prefabricated house (housing estate)	75	37.3	**Total**	**201**	**100.0**
Apartment building	40	19.9			
Home	75	37.3	**Method of childbirth**	**n**	**%**
Hostel	9	4.5	Vaginal birth	90	44.8
Other	2	1.0	Cesarean section (s.c.)	111	55.2
**Total**	**201**	**100.0**	**Total**	**201**	**100.0**
Note: n = absolute frequency; % - relative frequency					

© 2023 Abrmanová M. et al.
